# A Comparative Study of the Structural Dynamics of Four Terminal Uridylyl Transferases

**DOI:** 10.3390/genes8060166

**Published:** 2017-06-20

**Authors:** Kevin J. Cheng, Özlem Demir, Rommie E. Amaro

**Affiliations:** 1 Department of Chemistry & Biochemistry, University of California, San Diego, La Jolla, CA 92093, USA; kjcheng2@illinois.edu (K.J.C.); odemir@ucsd.edu (Ö.D.); 2 National Biomedical Computation Resource, University of California, San Diego, La Jolla, CA 92093, USA

**Keywords:** TUTases, terminal uridylyl transferases, POVME, electrostatics, pocket volume, pocket shape, RET1, RET2, MEAT1, TUT4, *Trypanosoma brucei*

## Abstract

African trypanosomiasis occurs in 36 countries in sub-Saharan Africa with 10,000 reported cases annually. No definitive remedy is currently available and if left untreated, the disease becomes fatal. Structural and biochemical studies of trypanosomal terminal uridylyl transferases (TUTases) demonstrated their functional role in extensive uridylate insertion/deletion of RNA. *Trypanosoma brucei* RNA Editing TUTase 1 (TbRET1) is involved in guide RNA 3’ end uridylation and maturation, while TbRET2 is responsible for U-insertion at RNA editing sites. Two additional TUTases called TbMEAT1 and TbTUT4 have also been reported to share similar function. TbRET1 and TbRET2 are essential enzymes for the parasite viability making them potential drug targets. For this study, we clustered molecular dynamics (MD) trajectories of four TUTases based on active site shape measured by Pocket Volume Measurer (POVME) program. Among the four TUTases, TbRET1 exhibited the largest average pocket volume, while TbMEAT1’s and TbTUT4’s active sites displayed the most flexibility. A side pocket was also identified within the active site in all TUTases with TbRET1 having the most pronounced. Our results indicate that TbRET1’s larger side pocket can be exploited to achieve selective inhibitor design as FTMap identifies it as a druggable pocket.

## 1. Introduction

Human African trypanosomiasis, or sleeping sickness, is endemic among the poorest countries in Central Africa. The causative agent of the disease is *Trypanosoma brucei*, an extracellular eukaryotic flagellate parasite [1,2]. According to the World Health Organization (WHO), only four drugs are registered to treat the parasite. However, complications may arise when considering toxicity and risk of parasite resistance [3]. The price of discovering and manufacturing drugs can be exorbitant; thus, alternative routes such as computational methods can help circumvent this problem [4].

*T. brucei* processes its mitochondrial premature mRNA into translatable mRNA through various post-transcriptional RNA editing processes that heavily rely on uridylate insertion/deletion [5]. The mitochondrial DNA is in the form of maxicircles and minicircles in which the sequence of pre-edited mRNAs is modified by a multi-protein complex called “the editosome” based on guide RNAs (gRNAs). These gRNAs are encoded by the minicircles and used as a template to modify pre-mRNA [6,7]. The partial complementarity of the gRNA with pre-mRNA provides sites in which uridylate nucleotides (U) are inserted or deleted. This process is repeated multiple times with different gRNAs resulting in translatable mRNA [5].

**Figure 1:**
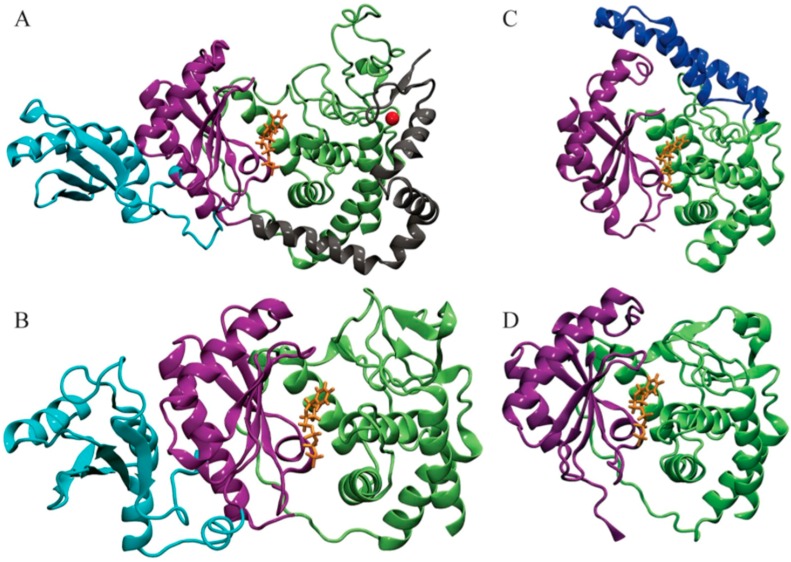
Structures of four *T. brucei* terminal uridylyl transferases (TUTases) shown in ribbons. (**A**) TbRET1; (**B**) TbRET2; (**C**) TbMEAT1; (**D**) TbTUT4. Enzymes are colored according to the domains: C-terminal domain (CTD) in green, N-terminal domain (NTD) in purple, middle domain (MiD) (RRM domain in TbRET1) in light blue, bridge domain (BD) in dark blue, and the zinc finger domain and connecting helix of TbRET1 in silver. uridine triphosphate (UTP) is shown in orange sticks, and Zn is shown as a red sphere.

Poly(A) polymerases (PAPs) and terminal uridylyl transferases (TUTases) are enzymes that catalyze the transfer of a nucleotide (adenylate and uridylate, respectively) to a hydroxyl group acceptor [8,9]. Both enzymes are members of a distinct nucleotidyl transferase superfamily called DNA polymerase beta, or Pol Beta, and share a signature helix-turn motif hG[G/S]X9-13Dh[D/E]h (X signifies amino acid any; h signifies hydrophobic amino acids) [10]. In most cases, a triad of acidic residues bind the two divalent metal ions required for catalysis. The chemical mechanism is also conserved throughout these polymerases, and consists of the 3′-hydroxyl group of the RNA primer attacking the α-phosphate of uridine triphosphate (UTP), releasing pyrophosphate without forming a covalent intermediate. One divalent metal cation (typically Mg^2+^) facilitates this reaction by lowering the affinity of 3′-hydroxyl for hydrogen, while the second metal cation helps to stabilize the pyrophosphate leaving group. Moreover, structural analysis of several different enzymes in the nucleotidyltransferase family demonstrates a conserved N-terminal polymerase domain topology: a five-stranded mixed beta-sheet flanked by two or three alpha-helices [10]. So far, the structures of only four *T. brucei* TUTases have been elucidated (Figure 1), and we will focus on and compare them in several aspects throughout this study.

Uridylylation catalyzed by mitochondrial RNA editing TUTase 1 (RET1) takes place at 3’-oligo U tail of gRNAs in addition to ribosomal RNAs (rRNAs) and some mRNAs. Moreover, RET1 has been shown to have high substrate affinity for single-stranded RNAs [11]. Studies of the recombinant protein from related parasite *Leishmania tarentolae* concluded that RET1 oligomerizes and can add hundreds of uridylates to unstructured RNA longer than 10 nucleotides. On the other hand, in vivo studies found that the U-tails found in both gRNAs and rRNAs were limited to approximately 15 nucleotides, indicating controlled processivity of this enzyme [11,12]. Investigations have shown the majority of RET1 proteins exist in a complex called the mitochondrial 3’ processome and is responsible for recognition, uridylation, and exonucleolytic processing of gRNA precursors along with U-tail addition of mature gRNA [12]. Crystal structures of *T. brucei* RET1 revealed the nucleotidyl transferase bi-domain as well as the RNA Recognition Motif (RRM) and functionally important C2H2 zinc finger domains (Figure 1A) [12].

RNA editing TUTase 2 (RET2), a TUTase also found in mitochondrial extract of *T. brucei*, is a subunit of the RNA-editing core complex (also known as “the editosome”) and is responsible for the U addition in mRNA specified by gRNA to precleaved double-stranded RNA [13]. Despite the differences in the biological role between TbRET1 and TbRET2, the two TUTases have 24% sequence similarity between their N-terminal catalytic (NTD) and C-terminal (CTD) domains [5,13]. In TbRET2, a 107-residue-long middle domain (MiD) is inserted between two beta sheets at the C-terminus of the NTD (Figure 1B). The MiD is a unique structural feature of TbRET2 compared to all other members of the nucleotidyl transferase superfamily. This domain extends into the solvent while interacting with the CTD. Moreover, the MiD shows a surface charge consistent with the potential role in RNA binding and is responsible for RET2-MP81 binding within the editosome complex. TbRET1 contains a similar domain called the RNA Recognition Motif (RRM) domain (Figure 1A) [12]. Although the RRM and MiD differ in primary structure, the domain positions are conserved and away from the catalytic site. The deletion of the MiD and RRM domains from TbRET2 and TbRET1, respectively, inhibits their enzyme functions, suggesting a role in protein folding or RNA substrate binding.

The smallest of the TUTases, TbTUT4, serves as a minimal model for this class of nucleotidyl transferases [8]. It is also an RNA-dependent U-specific nucleotidyl transferase that accepts exclusively single-stranded RNA as a substrate. It has 30% sequence identity with TbRET2; as a result, the NTD and CTD of TbTUT4 also form a spherically shaped bi-domain. In addition, TbTUT4 lacks any auxiliary domain like the ones found in TbRET1 (C2H2 Zn finger) or TbRET2 (MiD). TbRET2 and TbTUT4 differ in their biological functions, domain composition, sub-cellular localization, and RNA substrate specificity [8]. Mitochondrial TbRET2 is active on single-stranded and double-stranded RNA, while cytosolic TbTUT4 accepts exclusively single-stranded RNA as a substrate.

Another mitochondrial TUTase, *T. brucei* Mitochondrial Editosome-Like Complex Associated TUTase 1 (TbMEAT1), adds more complexity to the structure–function relationships when compared to the previously mentioned TUTases. Similar to TbRET2, TbMEAT1 associates with a protein complex resembling the editosome [13]. Within this complex, TbMEAT1 effectively replaces the U-insertion complex found in the editosome that consists of REL2, MP81, and RET2. Moreover, this enzyme is exclusively U-specific and capable of U insertion to both single-stranded and double-stranded RNA. In spite of TbMEAT1’s low sequence identity with TbRET2 (12%) and TbTUT4 (14%), it still adopts a bi-domain architecture forming a deep cleft containing an active site similar to the TUTases mentioned above (Figure 1) [13]. A unique domain in TbMEAT1 is the bridge domain (BD) that replaces the unstructured loop regions in TbRET2 and TbTUT4 [13]. Moreover, several active site residues that are common among trypanosomal TUTases are replaced in TbMEAT1 with either similar residues or residues with altered charge or polarity [13].

To summarize, all *T. brucei* TUTases mentioned above contain an NTD and CTD, sharing a bi-domain topology. This bi-domain confers a catalytic cleft with a common UTP-binding scheme for each enzyme. A divalent metal ion coordinates to the triphosphate moiety of UTP along with three invariant aspartates. Additionally, amino acids surrounding the uracil base provide further stabilization through water-mediated or direct hydrogen bonding in addition to base-stacking interactions provided by a Tyr (Phe in TbMEAT1) residue. TbRET1, TbRET2, and TbMEAT1 are found in the mitochondria unlike the cytosolic TbTUT4. All these enzymes, except TbTUT4, are shown to be essential for *T. brucei* viability (refer to Table 1 for overview of differences). The biological role of TbTUT4 has yet to be elucidated.

Although we understand the biochemical function of these biomolecules, a detailed description of how protein dynamics determines their biological role remains a scientific challenge [14,15,16]. Most enzymes undergo a series of conformational changes before and/or after they interact with their substrate. Experimental techniques used to probe these dynamical systems are generally limited in their spatiotemporal resolution, and most report ensemble averages. Computational simulations address this drawback by providing atomic scale spatial and femtosecond temporal resolution of molecular systems [16]. The data provided by computation improve our mechanistic understanding of conformational change, leading to insights not accessible by experiments.

In this work, we compared various aspects of active sites of these four TUTases to help future structure-based drug design efforts. The four TUTases we analyzed have a conserved catalytic pocket topology, making it an attractive target for trypanocide development. We utilized POVME, a computational tool that characterizes pocket shape and size of proteins. Using this program, we can quantitatively compare volume of binding pockets as a function of time that is not accessible to experiments. From the results, we quantitatively compare each TUTase and provide data to guide future drug discovery efforts.

## 2. Materials and Methods

### 2.1. Molecular Dynamics Simulations

#### 2.1.1. TbRET1

Three independent copies of 250 ns MD simulations were performed for UTP-bound TbRET1 (PDB ID: 5IDO). The final system consisted of 103,944 atoms. Snapshots of the system extracted every 10 ps were used to obtain a combined trajectory of 75,000 frames. The Amber FF14SB force field in the Amber 14 suite was used to parameterize the protein. Na and Cl ions were added to provide a salt concentration of 0.2 M. Production MD simulations were performed at 310 K. Further details for the system preparation and MD simulation protocol are described in previous work [7].

#### 2.1.2. TbRET2

Two independent copies of 50 ns MD simulations were performed for UTP-bound TbRET2 (PDB ID: 2B4V). The final system consisted of 65,384 atoms. Snapshots of the system extracted every 0.5 ps were used to obtain a combined trajectory of 200,000 frames. The Amber FF99SB force field was used to construct the topology files for each system. Chloride ions were added to each system for neutralization, and the production MD simulations were performed at 310 K. Further details for the system preparation and MD simulation protocol are described in previous work [17].

The MD simulations for TbMEAT1 (PDB ID: 3HJ1) and TbTUT4 (PDB ID: 2IKF) are performed following the same protocol as in TbRET2. The final systems consisted of 56,129 and 43,631 atoms for TbMEAT1 and TbTUT4, respectively.

### 2.2. Ensemble-Averaged Electrostatics

All electrostatic calculations were calculated using a VMD [18] plugin called DelEnsembleElec [19] interfacing with Delphi, a numerical Poisson-Boltzmann (PB) equation solving suite. An interior dielectric constant of 2, an exterior dielectric constant of 80, a grid scale of 1.0 (angstroms), and a probe radius of 1.4 Å to define the dielectric boundary were used. Convergence was achieved once the change in potential decreased below the threshold of 0.0001 kT/e. Differences in input parameter specifications are listed below.

#### 2.2.1. TbRET1

The ensemble-averaged electrostatic potential of UTP-bound *Tb*RET1 was calculated using 1500 frames extracted from 75,000 frames of MD simulations at regular 500 ps intervals. The electrostatic potential was calculated on a 115 × 115 × 115 static grid with a salt concentration of 0.2 M.

#### 2.2.2. TbRET2

The ensemble-averaged electrostatic potential of UTP-bound TbRET2 was calculated using 1000 frames extracted from 20,000 frames of MD simulations at regular 100 ps intervals. The electrostatic potential was calculated on a 111 × 111 × 111 static grid with a salt concentration of 0 M.

#### 2.2.3. TbMEAT1

The ensemble-averaged electrostatic potential of UTP-bound TbMEAT1 was calculated using 1000 frames extracted from 20,000 frames of MD simulations at regular 100 ps intervals. The electrostatic potential was calculated on an 85 × 85 × 85 static grid with a salt concentration of 0 M.

#### 2.2.4. TbTUT4

The ensemble-averaged electrostatic potential of UTP-bound TbTUT4 was calculated using 1000 frames extracted from 20,000 frames of MD simulations at regular 100 ps intervals. The electrostatic potential was calculated on an 85 × 85 × 85 static grid with a salt concentration of 0 M.

### 2.3. Clustering

The MD trajectory for each enzyme was clustered based on pocket shape using POVME 3.0 [20,21], which is a program that uses a single inclusion sphere encompassing the binding cleft formed by the NTD and CTD to quantify the shape of this region. The same inclusion sphere was used for all TUTases after superimposing each one using the VMD plugin “multiseq” [22] and aligning their trajectories to their first frames. The POVME algorithm calculates the pocket volume by subtracting the volume occupied by the protein atoms in each frame from the inclusion sphere volume.

## 3. Results

### 3.1. POVME Analysis

The active site volume for each enzyme during MD simulations was calculated using POVME 3.0. TbRET1 was found to have the greatest average pocket volume, followed by TbRET2, TbMEAT1, and TbTUT4, respectively (Table 2). Standard deviation in pocket volume was greatest for TbTUT4, followed by TbMEAT1, TbRET1, and TbRET2, respectively, indicating that TbTUT4’s pocket volume fluctuated the most (Table 2). This also suggests that TbTUT4’s active site shape changes the most among the four enzymes as volume changes directly reflect pocket shape changes. TbRET2 and TbRET1, on the other hand, had relatively more rigid active sites.

**Figure 2:**
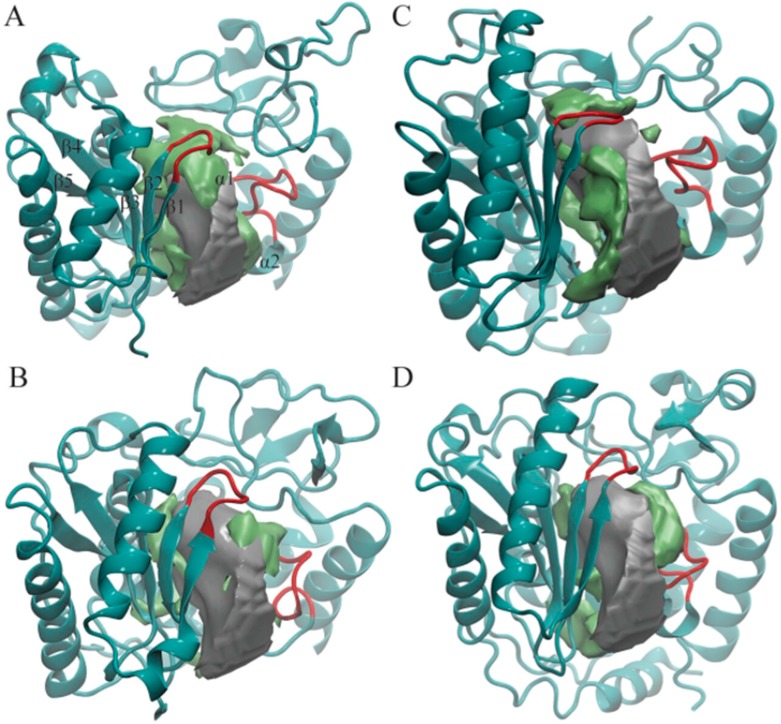
Pocket shapes of the most-populated four cluster centroids: (**A**) TbRET1; (**B**) TbRET2; (**C**) TbMEAT1; (**D**) TbTUT4. Only NTD and CTD are shown in cyan ribbons in each enzyme for clarity. The average pocket shape of all enzymes is shown as a silver surface, and the additional region opening only in that cluster are shown as a green surface. The two loops we found to modulate the pocket shape are highlighted with red. Beta-sheets and alpha-helices close to the active site are labeled on TbRET1 only (α1 labeled, but not visible).

POVME 3.0 can also cluster MD trajectories based on active site shapes and sizes using a k-means clustering algorithm. For this work, we generated a total of 19 clusters by clustering the combined trajectory of all four enzymes. The average volume calculated for cluster members and its standard deviation is depicted for each cluster in Table 3. We observed that each cluster contained MD snapshots of only one enzyme, suggesting that the active site shapes of these enzymes are distinctive from one another (Supplementary Materials Figure S1). A total of 8, 6, 3, and 2 clusters were generated consisting of active site conformations solely from TbTUT4, TbMEAT1, TbRET1, and TbRET2, respectively (Table 3). In addition, there is a positive correlation between the standard deviation of pocket volumes and the number of clusters each enzyme generated. For example, TbTUT4 displayed the greatest volume deviation (Table 2) and also had the greatest number of clusters (Table 3). Likewise, TbRET2, which had the lowest standard deviation in pocket volume (Table 2), had the least number of clusters (Table 3). The greater the standard deviation in volumes is, the more likely it is that the enzyme will explore different conformations with distinct active site shapes. Based on the visual observations of each cluster centroid, the two loops depicted in red in Figure 2 are the most flexible regions modulating TUTase active site shape and volume.

**Figure 3:**
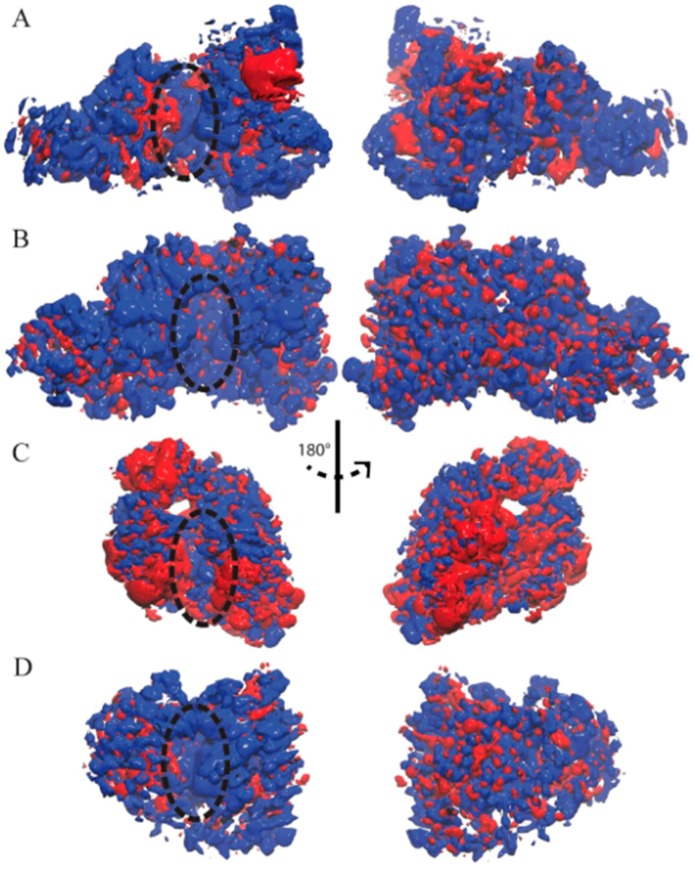
Ensemble-averaged electrostatics of (**A**) TbRET1; (**B**) TbRET2; (**C**) TbMEAT1; (**D**) TbTUT4. The exact same orientations shown in Figure 1 are used to demonstrate the electrostatic potential surface on the left, and the rear views are shown on the right. The blue patches represent areas of positive electrostatic potential, and the red patches represent areas of negative potential. Active site is designated with a dashed oval.

**Figure 4:**
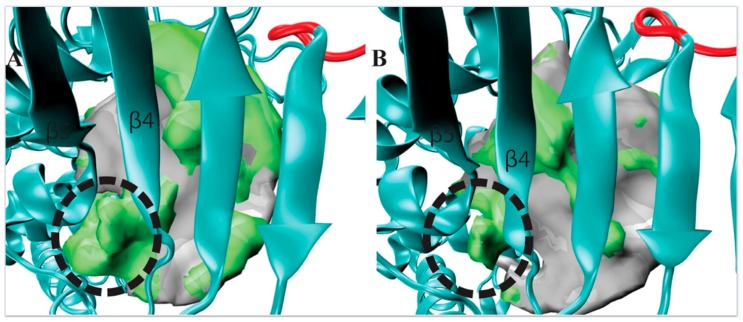
A side pocket was identified from visualizing our POVME results for the centroid of Cluster 1 (TbRET1, Panel **A**). The same region is compared with the centroid of Cluster 2 (TbRET2 Panel **B**) to demonstrate the pronounced pocket volume increase. Additional regions opening only in that cluster are shown as a green surface relative to the average pocket volume. The side pocket is located between β4 and β5.

In order to capture the most salient aspects of our large POVME dataset, we compared the cluster centroids. The k-means clustering algorithm generates centroids, each of which is an average of all data points (in this case, snapshots from the trajectory) within each cluster. Each centroid can be thought of as a frame that best represents the cluster to which it belongs. We used VMD to visually analyze all 19 POVME-generated centroids that showed pocket volume increases relative to the average trajectory volume (Figure 2). TbRET1, TbRET2, and TbTUT4 (Figure 3) revealed a side pocket opening adjacent to the loop between β4 and β5 (Figure 4). Based on our observations, this pocket opening is primarily caused by the flexibility of this loop rather than the change of side chains’ rotameric states. Notably, the Cα of Ser 65 in TbTUT4, which participates in UTP binding, showed a translation of at least 2.73 Å. Moreover, TbRET2 formed an additional unique side pocket positioned between α1 and α2 (Figure 2B). This side pocket results from the flexibility of the loop connecting these two helices as can be observed by the 4.73 Å displacement of the Cα of Ser 312 in Centroid 2 compared to Centroid 13. TbMEAT1 showed a relatively larger solvent accessible surface between β3 and β4 compared to the other TUTases (Figure 2C). Particularly, the Cα of His 63 demonstrated a translation of at least 3.44 Å.

### 3.2. Ensemble-Averaged Electrostatics

The ensemble-averaged electrostatic potential was computed for each enzyme based on the UTP-bound MD trajectories (Figure 3). The electrostatic potential of TbRET1 demonstrated more positive electrostatic patches near the active site (Figure 3A, left panel). Additionally, a small negative patch was observed near β3 and β4 and a large negative patch was found close to the Zn finger domain. The backside of the protein showed more positive patches encompassing the NTD and RRM (Figure 3A, right panel).

The electrostatic potential for TbRET2 displayed a more uniform positive patch around the periphery of the active site and encompasses most of the front side of the protein (Figure 3B, left panel). Moreover, the backside of the protein consisted of evenly distributed positive and negative electrostatic patches.

TbMEAT1 demonstrated a negative electrostatic patch around the NTD and a positive patch near the active site within the CTD (Figure 3C, left panel). Additionally, a pronounced negative patch was found within the middle domain at the N-terminal side of the enzyme.

TbTUT4 predominantly displayed positive electrostatic potential on the front face of the enzyme (Figure 3D, left panel). On the other hand, the backside revealed an even distribution of positive and negative electrostatic patches (Figure 3D, right panel).

For all *T. brucei* TUTases here, positive electrostatic regions are mostly found near the active site, making them attractive for RNA substrate binding.

## 4. Discussion

MD simulations and POVME analysis provided a means to quantitatively compare how the active site shape and volume differ for each TUTase. Upon inspecting different cluster centroids, we found that rigid body side chain translations rather than side chain rotations account mostly for the active site changes.

The clustering algorithm has generated 8 and 6 clusters for TbTUT4 and TbMEAT1, while only 3 and 2 clusters for TbRET1 and TbRET2, respectively. This suggests that TbTUT4’s and TbMEAT1’s trajectories sample relatively more diverse structures that modify the shape of the active site. Several factors contribute to TbTUT4’s pocket shape heterogeneity. Particularly, the loop region connecting beta sheets β1 and β2 (Figure 2D) and the flexible region connecting β4 and β5. The flexible loop which consists of residues 275 to 291 within the CTD also exhibits large displacements when comparing all centroids of TbTUT4. On the other hand, TbMEAT1 exhibited an increasing solvent accessible surface due to separation between β3 and β4. When comparing the transiently open active site regions (designated by green surfaces) of the TUTases in Figure 2, TbMEAT1 contains a unique extended volume region extending between these two beta sheets. The loop connecting α1 and α2 also plays a role in modulating the active site volume by a significant translation towards the center of the pocket as observed in Cluster 15. This is quantitatively supported by the fact that Cluster 15 has the lowest average pocket volume compared to the other TbMEAT1 clusters. In TbMEAT1, the loop connecting β1 and β2 (Figure 2C) did not show much flexibility compared to the other TUTases.

In TbRET1, the loop joining β1 and β2 (Figure 2A) is flexible enough to modulate the volume of the active site. We also witnessed a side pocket forming between β4 and β5 in TbRET1 (Cluster 5). The fact that TbRET1 trajectory only generated three clusters implies that the enzyme samples a less diverse conformational space, indicating that the side pocket formation is not a rare event. This side pocket can be exploited for developing more selective drugs that are potent towards TbRET1. Moreover, this enzyme exhibits the greatest pocket volume, making it an even more attractive target for inhibitor design. It should also be noted that TbRET1 was simulated longer than the other enzymes only because of the availability of resources. In order to verify that the larger volume observed was not due to the longer simulation time, we calculated the average volume of the first 50 ns of TbRET1 MD copies instead of the entire 250 ns. It was found to be 2507.03 ± 137.44, still the greatest among the four TUTases.

TbRET2 shows the least number of clusters implying the least conformational heterogeneity. A side pocket can be seen forming between α1 and α1. Similarly, the same side pocket seen in TbRET1 is observed in TbRET2 between β4 and β5 but with a smaller volume.

The observed side pocket in TbRET1 offers an opportunity to design specific and potent inhibitors (Figure 4A) since this centroid belongs to the most populated cluster (Cluster 1) and contains 76% of TbRET1 simulation data. This suggests that most conformations that the enzyme samples contain this additional cavity.

Additionally, we inspected the residues surrounding this side pocket region and compared the different centroids. Our POVME data suggests that this pocket is formed not because of side chain rotations, but backbone translation. For example, the largest side chain group within this loop region (residues 303 to 310), the indole group of Trp 305, showed a displacement of 2.79 Å between the two nitrogens on the pyrrole rings. We also evaluated the druggability of this site using FTMap [23]. FTMap program identifies regions that have the most energetically favorable binding site by flooding the surface of the protein of the molecule and computing the interaction energy. Probe molecules were found binding this region, further supporting the idea that future efforts should focus on exploiting this region for inhibitor design.

## 5. Conclusions

In this study, we compared four TUTases responsible for RNA uridylation that play a role mitochondrial gene regulation in Trypanosomes. We explored their dynamics utilizing MD simulations and monitored how their active site volume and shape changes. This was achieved using POVME, a program that quantifies the volume and assists the visualization of active site volumes. We found that TbRET1 displayed the greatest average volume followed by TbRET2, TbMEAT1, and TbTUT4, respectively.

We also found that TbMEAT1 and TbTUT4 active sites exhibit more conformational diversity than TbRET1 and TbRET2 based on our clustering data. In general, we found that the two flexible loops flanking the active site (Figure 2) modulate the active sites of these enzymes.

A side pocket between β4 and β5 can be seen forming for all the TUTases, but TbRET1 demonstrated the most spacious pocket. This suggests a unique inhibitor design possibility for TbRET1 that does not appear to exist in the other TUTases, and suggests that TbRET1 may be a better target for orthosteric ligand design because of its larger pocket volume. Additionally, an additional side pocket was found in TbRET1 that can be exploited to achieve TbRET1 selectivity of inhibitors. This is further supported by FTMap analysis (Supplementary Materials Figure S2), showing the side pocket is druggable.

## Figures and Tables

**Table 1:** Properties of the four TUTase enzymes in this work.

Enzyme	In Complex With	Domains	Substrates	Ref.
RET1	Mitochondrial 3’ processome (MPsome)	C2H2 Zn Finger, CTD, NTD, RRM	UTP, high affinity for ssRNA	[12]
RET2	RNA Editing Core Complex (Editosome)	Middle Domain, CTD, NTD	UTP, ssRNA, dsRNA (needs gRNAs)	[5]
MEAT1	Resembles editosome	Bridge Domain, CTD, NTD	Exclusively U Specific, ssRNA, dsRNA	[13]
TUT4	Unknown	NTD, CTD (minimal TUTase)	Prefers UTP and exclusively single-stranded RNA as a primer	[8]

**Table 2:** The average pocket volume and its standard deviation over the specified number of frames for each enzyme.

Enzyme	Average Volume/Å^3^	Standard Deviation	Number of Frames
TbRET1	2434	194	1500
TbRET2	2061	145	1000
TbMEAT1	1831	247	1000
TbTUT4	1768	316	1000

**Table 3:** The average pocket volume calculated for each cluster. Each cluster was found to contain only one enzyme listed in the second column, suggesting that the pocket volume and shape is distinct for each TUTase.

Cluster	Enzyme	Average Volume/Å^3^	Standard Deviation
1	TbRET1	2508	147
2	TbRET2	2073	134
3	TbMEAT1	2071	143
4	TbTUT4	1750	83
5	TbRET1	2195	116
6	TbTUT4	2175	132
7	TbMEAT1	1661	90
8	TbMEAT1	1810	102
9	TbTUT4	1506	90
10	TbMEAT1	1535	91
11	TbTUT4	1191	65
12	TbTUT4	1393	97
13	TbRET2	1772	95
14	TbTUT4	1610	75
15	TbMEAT1	1318	52
16	TbTUT4	1749	80
17	TbMEAT1	1711	48
18	TbRET1	1848	4
19	TbTUT4	1620	0

## Supplementary Materials

The following are available online at www.mdpi.com/2073-4425/8/6/166/s1. Figure S1: A plot showing the number of frames within each cluster. The cluster labels start from 0 instead of 1. The abscissa designates the frame index for each protein. Cluster 17 and Cluster 18 have minimal frames (3 and 1 respectively), Figure S2: The FTMap probe shown in VDW representation is found precisely in the side pocket region previously mentioned in Figure 4A. These results further support our hypothesis that this region is druggable.
