# Supplementary figures and images for: A Comparative Study of the Structural Dynamics of Four Terminal Uridylyl Transferases
Source: Genes (Basel). 2017 Jun 20;8(6):166. doi: 10.3390/genes8060166 (PMC5485530; doi:10.3390/genes8060166)

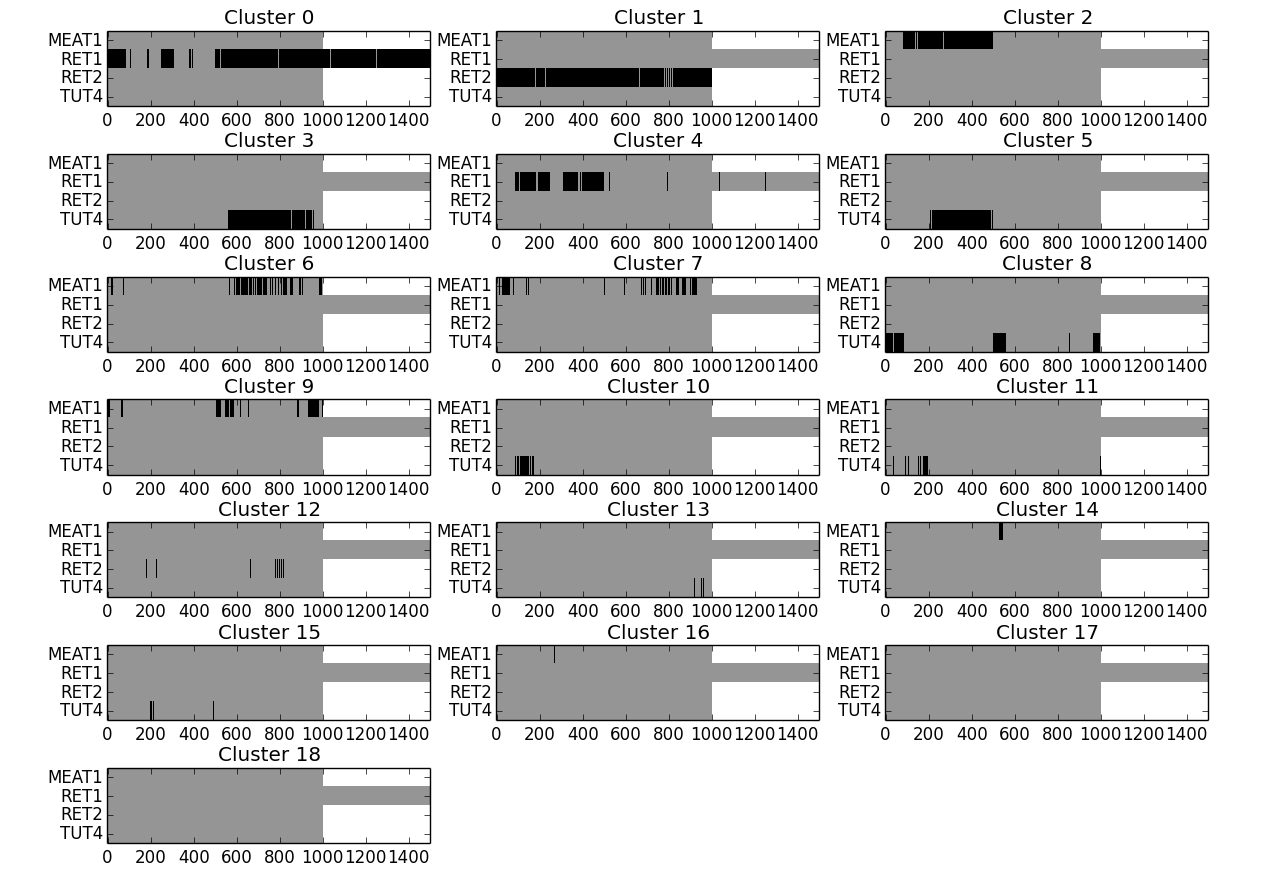

Supplement: Supplementary file 1 [file genes-08-00166-s001.zip › FigS1_ClusterMembers_Supplementary.png]

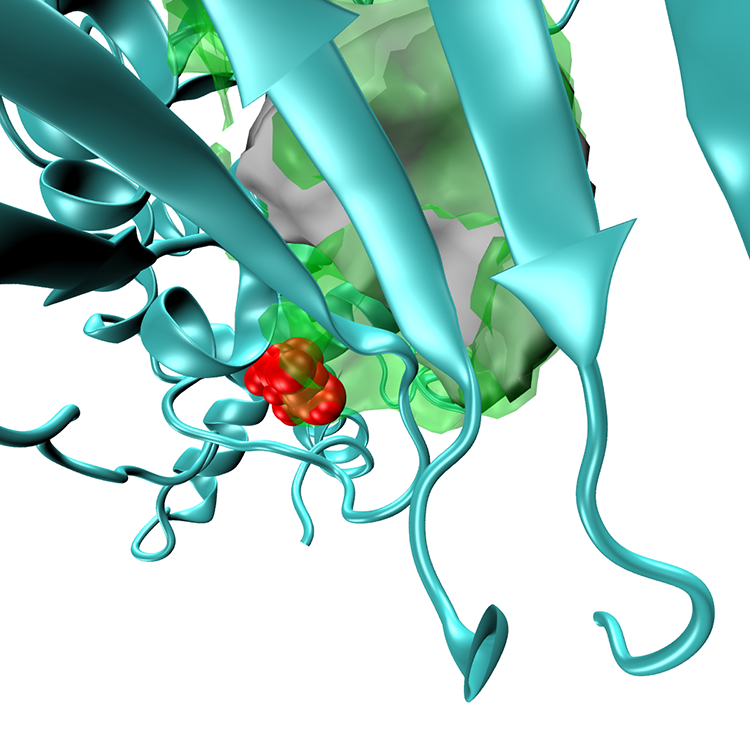

Supplement: Supplementary file 1 [file genes-08-00166-s001.zip › FigS2_RET1_SidePocket_withFTMAPProbe.tif]
